# Poor oral health influences head and neck cancer patient survival: an International Head and Neck Cancer Epidemiology Consortium pooled analysis

**DOI:** 10.1093/jnci/djad156

**Published:** 2023-09-19

**Authors:** Jason Tasoulas, Douglas R Farquhar, Siddharth Sheth, Trevor Hackman, Wendell G Yarbrough, Chris B Agala, Alzina Koric, Luca Giraldi, Eleonora Fabianova, Jolanta Lissowska, Beata Świątkowska, Marta Vilensky, Victor Wünsch-Filho, Marcos Brasilino de Carvalho, Rossana Verónica Mendoza López, Ivana Holcátová, Diego Serraino, Jerry Polesel, Cristina Canova, Lorenzo Richiardi, Jose P Zevallos, Andy Ness, Miranda Pring, Steve J Thomas, Tom Dudding, Yuan-Chin Amy Lee, Mia Hashibe, Paolo Boffetta, Andrew F Olshan, Kimon Divaris, Antonio L Amelio

**Affiliations:** Lineberger Comprehensive Cancer Center, The University of North Carolina at Chapel Hill, Chapel Hill, NC, USA; Department of Otolaryngology/Head and Neck Surgery, University of North Carolina at Chapel Hill, Chapel Hill, NC, USA; Division of Oral and Craniofacial Health Sciences, Adams School of Dentistry, University of North Carolina at Chapel Hill, Chapel Hill, NC, USA; Department of Otolaryngology/Head and Neck Surgery, University of North Carolina at Chapel Hill, Chapel Hill, NC, USA; Division of Hematology/Oncology, Department of Medicine, The University of North Carolina at Chapel Hill, Chapel Hill, NC, USA; Department of Otolaryngology/Head and Neck Surgery, University of North Carolina at Chapel Hill, Chapel Hill, NC, USA; Lineberger Comprehensive Cancer Center, The University of North Carolina at Chapel Hill, Chapel Hill, NC, USA; Department of Otolaryngology/Head and Neck Surgery, University of North Carolina at Chapel Hill, Chapel Hill, NC, USA; Department of Pathology and Laboratory Medicine, School of Medicine, University of North Carolina at Chapel Hill, Chapel Hill, NC, USA; Department of Surgery, University of North Carolina at Chapel Hill, Chapel Hill, NC, USA; Division of Public Health, Department of Family and Preventive Medicine and Huntsman Cancer Institute, University of Utah School of Medicine, Salt Lake City, UT, USA; Section of Hygiene, University Department of Life Sciences and Public Health, Università Cattolica del Sacro Cuore, Rome, Italy; Regional Authority of Public Health, Banska Bystrica, Slovakia; Department of Cancer Epidemiology and Prevention, M. Sklodowska-Curie National Research Institute of Oncology, Warsaw, Poland; Department of Environmental Epidemiology, Nofer Institute of Occupational Medicine, Lodz, Poland; Institute of Oncology Angel H. Roffo, University of Buenos Aires, Buenos Aires, Argentina; Epidemiology Department, School of Public Health, University of São Paulo, São Paulo, Brazil; Oncocentro Foundation of São Paulo, São Paulo, Brazil; Department of Head and Neck, Heliopolis Hospital, São Paulo, Brazil; Cancer Institute of the State of São Paulo (ICESP), São Paulo, Brazil; Institute of Hygiene and Epidemiology, Charles University in Prague, Prague, Czech Republic; Unit of Cancer Epidemiology, Centro di Riferimento Oncologico di Aviano (CRO) IRCCS, Aviano, Italy; Unit of Cancer Epidemiology, Centro di Riferimento Oncologico di Aviano (CRO) IRCCS, Aviano, Italy; University of Padua, Padova, Italy; Department of Medical Sciences, University of Turin, Turin, Italy; Department of Otolaryngology/Head and Neck Surgery, University of Pittsburgh, PA, USA; Bristol Dental School, University of Bristol, Bristol, UK; Bristol Dental School, University of Bristol, Bristol, UK; Bristol Dental School, University of Bristol, Bristol, UK; Bristol Dental School, University of Bristol, Bristol, UK; Division of Public Health, Department of Family and Preventive Medicine and Huntsman Cancer Institute, University of Utah School of Medicine, Salt Lake City, UT, USA; Division of Public Health, Department of Family and Preventive Medicine and Huntsman Cancer Institute, University of Utah School of Medicine, Salt Lake City, UT, USA; Stony Brook Cancer Center, Department of Family, Population and Preventive Medicine, Stony Brook University, Stony Brook, NY, USA; Department of Medical and Surgical Sciences, University of Bologna Italy; Department of Otolaryngology/Head and Neck Surgery, University of North Carolina at Chapel Hill, Chapel Hill, NC, USA; Department of Epidemiology, Gillings School of Global Public Health, The University of North Carolina at Chapel Hill, Chapel Hill, NC, USA; Department of Epidemiology, Gillings School of Global Public Health, The University of North Carolina at Chapel Hill, Chapel Hill, NC, USA; Division of Pediatric and Public Health, Adams School of Dentistry, University of North Carolina at Chapel Hill, Chapel Hill, NC, USA; Lineberger Comprehensive Cancer Center, The University of North Carolina at Chapel Hill, Chapel Hill, NC, USA; Department of Otolaryngology/Head and Neck Surgery, University of North Carolina at Chapel Hill, Chapel Hill, NC, USA; Division of Oral and Craniofacial Health Sciences, Adams School of Dentistry, University of North Carolina at Chapel Hill, Chapel Hill, NC, USA; Department of Tumor Biology, H. Lee Moffitt Cancer Center & Research Institute, Tampa, FL, USA; Department of Head and Neck-Endocrine Oncology, H. Lee Moffitt Cancer Center & Research Institute, Tampa, FL, USA

## Abstract

**Background:**

Poor oral health has been identified as a prognostic factor potentially affecting the survival of patients with head and neck squamous cell carcinoma. However, evidence to date supporting this association has emanated from studies based on single cohorts with small-to-modest sample sizes.

**Methods:**

Pooled analysis of 2449 head and neck squamous cell carcinoma participants from 4 studies of the International Head and Neck Cancer Epidemiology Consortium included data on periodontal disease, tooth brushing frequency, mouthwash use, numbers of natural teeth, and dental visits over the 10 years prior to diagnosis. Multivariable generalized linear regression models were used and adjusted for age, sex, race, geographic region, tumor site, tumor-node-metastasis stage, treatment modality, education, and smoking to estimate risk ratios (RR) of associations between measures of oral health and overall survival.

**Results:**

Remaining natural teeth (10-19 teeth: RR = 0.81, 95% confidence interval [CI] = 0.69 to 0.95; ≥20 teeth: RR = 0.88, 95% CI = 0.78 to 0.99) and frequent dental visits (>5 visits: RR = 0.77, 95% CI = 0.66 to 0.91) were associated with better overall survival. The inverse association with natural teeth was most pronounced among patients with hypopharyngeal and/or laryngeal, and not otherwise specified head and neck squamous cell carcinoma. The association with dental visits was most pronounced among patients with oropharyngeal head and neck squamous cell carcinoma. Patient-reported gingival bleeding, tooth brushing, and report of ever use of mouthwash were not associated with overall survival.

**Conclusions:**

Good oral health as defined by maintenance of the natural dentition and frequent dental visits appears to be associated with improved overall survival among head and neck squamous cell carcinoma patients.

Head and neck squamous cell carcinoma is the sixth most common malignancy worldwide, with 878 348 newly diagnosed patient cases in 2020 (1). Although survival has improved over the past decades, head and neck squamous cell carcinoma remains one of the most lethal malignancies worldwide, with 444 347 reported deaths in 2020 (1). Variation in global head and neck squamous cell carcinoma incidence (2) reflects differences in the distribution of known risk factors including smoking and tobacco exposure (3), alcohol (4), human papillomavirus (HPV) (5), and low socioeconomic status (6,7). Importantly, these risk factors have also been associated with survival differences of head and neck squamous cell carcinoma patients (8-11).

Poor oral health has been reported as an independent risk factor for head and neck squamous cell carcinoma (12). Specifically, measures of poor oral health including tooth loss, periodontal disease, infrequent tooth brushing, and lack of dental visits have been associated with weak to moderate increases in head and neck squamous cell carcinoma risk (12,13). Although the mechanisms underlying these associations remain unclear, chronic trauma (14), oral inflammation (15), and alterations in the oral microbiome (16) have been proposed. For example, oxidative stress is found in periodontal inflammation (17) and epithelial mutagenesis (18) and could link oral inflammation with cancer initiation and progression. Also, *Fusobacterium* species known to be increased in oral squamous cell carcinoma (19-21) were recently reported to induce the upregulation of programmed cell death ligand 1 and extracellular signal-regulated kinase 1 (ERK1) pathway signaling to the MYC proto-oncogene in head and neck squamous cell carcinoma and thus potentially affect tumor biology and treatment responses (22).

Notably, data on the impact of oral health on head and neck squamous cell carcinoma survival are currently limited and emanate from single cohorts with relatively small to moderate sample sizes (23-25). The definition of oral hygiene varies by study, and its association between poor oral hygiene and survival is inconsistent (23,25). In this study, we sought to add to the evidence base of oral health and determinants of overall survival in head and neck squamous cell carcinoma patients by analyzing demographic, clinicopathologic, oral health, treatment, and survival data from epidemiologic studies participating in the International Head and Neck Cancer Epidemiology (INHANCE) Consortium. This study reports the results of the largest pooled analysis of oral health and head and neck squamous cell carcinoma patient overall survival performed to date.

## Methods

### Participants and data

The pooled cohort of the INHANCE Consortium studies comprised 10 042 participants with head and neck cancer from 10 INHANCE case-control studies conducted in North America (Seattle, Washington, USA; Los Angeles, California, USA; HOTSPOT, CHANCE/North Carolina, USA), South America (Sao Paulo 1, Sao Paulo 2, Latin America), and Europe (Central Europe, Western Europe, Head and Neck 5000 [HN5000]). Data were obtained via self-reported questionnaires and harmonized as previously described (12,26,27). Study participants provided written informed consent, and studies were approved by the institutional review board at each institution involved (28).

We used available information for participants’ age, sex, race, smoking status, education level, year of diagnosis, tumor-node-metastasis (TNM) stage (American Joint Committee on Cancer 7th edition), tumor site, and treatment modality. Age was measured as a continuous value (range = 21-92 years), sex was binary (male, female), race was categorical (Asian and Pacific Islanders, Black, Brazilian, Others, White), geographic region was categorical (North America, South America, Europe), tumor site was categorical (oral cavity, oropharynx, hypopharynx and/or larynx, head and neck squamous cell carcinoma not otherwise specified [NOS]), TNM stage was categorical (I-IV), treatment was categorical, and tumors located in overlapping regions within the head and neck were termed as head and neck squamous cell carcinoma NOS, education was categorical (less than junior high school, some high school, high school graduate, technical school, college graduate or above), and smoking was categorical (current, former, never smokers).

**Figure 1.**
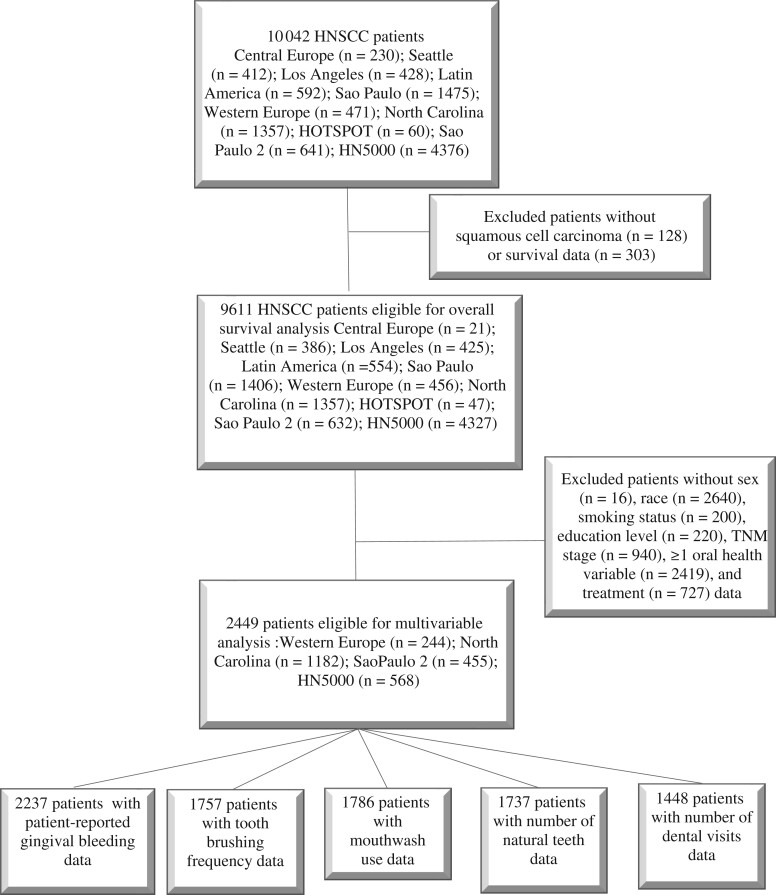
Flowchart of head and neck squamous cell carcinoma patient selection from the International Head and Neck Cancer Epidemiology Consortium for inclusion in the survival analysis. HN5000 = Head and Neck 5000; HNSCC = head and neck squamous cell carcinoma; HOTSPOT = Human Papillomavirus (HPV) Oral Transmission Study in Partners Over Time.

Information on HPV status was available only for 1 study and thus was not considered in this pooled analysis. Alcohol consumption information was available only for a subset (568 of 2449; 23%) of participants. Educational level was used as surrogate for socioeconomic status. Participants diagnosed with a histologic type other than head and neck squamous cell carcinoma (n = 128), as well as those with missing data on race (n = 2640), TNM stage (n = 940), treatment modality (n = 727), survival (n = 303), education level (n = 220), smoking status (n = 200), sex (n = 16), and availability of at least 1 health variable (ie, self-reported gingival bleeding, toothbrushing frequency, mouthwash use, number of remaining natural teeth, dental visits during the past 10 years; n = 2419) were excluded (see Figure 1). A total of 2449 eligible head and neck squamous cell carcinoma patients defined the analytical sample for survival analyses.

Information on patients’ oral health was available for self-reported gingival bleeding, tooth brushing frequency, mouthwash use, number of natural teeth, and number of dental visits during the past 10 years. Oral health measures were categorized according to definitions employed in the contributing studies. Whenever there was an incompatibility between individual study definitions, we used the definition that allowed the inclusion of the maximum number of participants. Specifically, self-reported gingival bleeding (yes, no), toothbrushing frequency (brushing <1 per day or brushing ≥1 times per day), and mouthwash use (yes, no) were dichotomized. The numbers of remaining natural teeth (≥20 teeth, 10-19 teeth, 1-9 teeth and no natural teeth) and dental visits during the past 10 years (no dental visits, 1-5 visits, and >5 visits) were treated as categorical variables. All measures referred to a time frame prior to cancer diagnosis, as previously described (10,12,29).

### Statistical analysis

Summary statistics for demographic characteristics were compared using frequencies and means. χ^2^ tests were used to compare dental visits during the past 10 years and early (stages I and II) vs late (stages III and IV) head and neck squamous cell carcinoma diagnosis. Survival time in years was compared using medians and Kaplan–Meier curves. We attempted to estimate hazard ratios (HRs) for various demographic and clinical predictors using Cox proportional hazards regression and thereafter tested for the proportionality assumption and discovered the assumption was violated. Therefore, we estimated the 5-year and 10-year survival functions using the Kaplan–Meir curves and examined between-group differences using the Wilcoxon–Breslow–Gehan test. Crude 5- and 10-year survival rates were estimated to compare survival based on key oral health variables including gingival bleeding, tooth brushing frequency, mouthwash use, missing teeth, and dental visits during the past 10 years. A generalized linear regression model using a log-link function with Poisson family and robust variance was used to estimate associations between measures of oral health and survival. The model included age, sex, race, smoking status, education level, TNM stage, tumor site, and treatment modality. Incidence rate ratios (rate ratio [RR]) and corresponding 95% confidence intervals (CI) of measures of oral health with overall survival were estimated. Age, sex, race, TNM stage, tumor site, and treatment modality were selected a priori for the models based on prior knowledge in the literature (12,21,24), whereas smoking and education were included as plausible confounders of the association between oral health survival. Analyses were carried out using Stata 16.1 (StataCorp LP, College Station, TX, USA).

## Results

The analytical sample included 2449 patients from the North Carolina (48%), HN5000 (23%), Sao Paulo 2 (19%), and Western Europe (10%) studies (Table 1). Participants were predominantly male (78%), had a mean age of 59.8 years, were diagnosed between 2002 and 2014, and were subsequently followed for a median 4.2 years (patient reported gingival bleeding, 4 years; toothbrushing frequency, 4 years; mouthwash use, 3.6 years; number of natural teeth, 3.4; number of dental visits, 4.8 years) (Table 2). Most participants were current (52%) or former (33%) smokers and of low educational attainment (53% attended some high school or less).

**Table 1.** Samples of patients with oral health measures in International Head and Neck Cancer Epidemiology contributing studies, overall and according to tumor site^a^

	Entire sample	Oral cavity	Oropharynx	Hypopharynx and/or Larynx	Head and neck squamous cell carcinoma NOS
	No. (col. %)	No. (row %)	No. (row %)	No. (row %)	No. (row %)
**All participants**	**2449 (100)**	**621 (25)**	**744 (30)**	**865 (35)**	**219 (9)**

North Carolina	1182 (48)	167 (14)	324 (27)	476 (40)	215 (18)
Sao Paulo 2	455 (19)	202 (44)	110 (24)	143 (31)	0 (0)
Western Europe	244 (10)	84 (34)	62 (25)	94 (39)	4 (2)
Head and Neck 5000	568 (23)	168 (30)	248 (44)	152 (27)	0 (0)

a Col = column; NOS = not otherwise specified.

**Table 2.** Demographic characteristics, smoking status, and oral health measures of head and neck squamous cell carcinoma patients from the International Head and Neck Cancer Epidemiology Consortium studies in the entire sample and according to tumor site

	Entire sample	Oral cavity	Oropharynx	Hypopharynx and/or Larynx	Head and neck squamous cell carcinoma NOS
Characteristics	No. (col. %)	No. (col. %)	No. (col. %)	No. (col. %)	No. (col. %)
Sex					
Male	1921 (78)	433 (70)	606 (81)	726 (84)	156 (71)
Female	528 (22)	188 (30)	138 (19)	139 (16)	63 (29)
Age, mean (SD), y	59.8 (10.5)	60.3 (11.4)	57.3 (9.4)	61.9 (10.0)	58.2 (11.2)
Race					
Asian and Pacific Islanders	16 (1)	9 (1)	3 (0)	4 (0)	0 (0)
Black	320 (13)	58 (9)	81 (11)	133 (15)	48 (22)
Brazilian^a^	2 (9)	0 (0)	1 (0)	1 (0)	0 (0)
Others^b^	138 (6)	47 (8)	41 (6)	46 (5)	4 (2)
White	1973 (81)	507 (82)	618 (83)	681 (79)	167 (76)
Education level					
No more than junior high school	333 (14)	117 (19)	79 (11)	125 (14)	12 (5)
Some high school	945 (39)	262 (42)	272 (37)	362 (42)	49 (22)
High school graduate	466 (19)	107 (17)	122 (16)	177 (20)	60 (27)
Technical school	431 (18)	78 (13)	154 (21)	138 (16)	61 (28)
At least college graduate	274 (11)	57 (9)	117 (16)	63 (7)	37 (17)
Smoking status					
Never smoker	355 (14)	105 (17)	156 (21)	43 (5)	51 (26)
Former smoker	816 (33)	190 (31)	285 (38)	290 (34)	51 (26)
Current smoker	1278 (52)	326 (52)	303 (41)	532 (62)	117 (53)
Gum bleeding					
Yes	647 (29)	159 (29)	175 (25)	246 (31)	67 (31)
No	1590 (71)	386 (71)	512 (75)	541 (69)	151 (69)
Missing	212	76	57	78	1
Tooth brushing					
<1 time/day	1224 (70)	200 (50)	330 (70)	486 (73)	208 (95)
≥1 time/day	533 (30)	202 (50)	141 (30)	180 (27)	10 (5)
Missing	692	219	273	199	1
Mouthwash use					
Yes	824 (46)	169 (40)	211 (45)	313 (46)	131 (60)
No	962 (54)	249 (60)	263 (55)	364 (54)	86 (40)
Missing	663	203	270	188	2
Dental visits in last 10 y					
0	305 (21)	86 (22)	68 (17)	124 (25)	27 (18)
1-5	661 (46)	225 (57)	168 (42)	231 (46)	37 (24)
>5	482 (33)	81 (21)	162 (41)	151 (30)	88 (58)
Missing	1001	229	346	359	67
Natural teeth					
0	395 (23)	83 (25)	90 (16)	180 (29)	42 (20)
1-9	166 (10)	28 (8)	46 (8)	77 (12)	15 (7)
10-19	312 (18)	64 (19)	119 (21)	106 (17)	23 (11)
≥20	864 (50)	157 (47)	313 (55)	261 (42)	133 (62)
Missing	712	289	176	241	6
TNM stage					
I	458 (19)	140 (23)	35 (5)	233 (27)	50 (23)
II	407 (17)	118 (19)	64 (9)	178 (21)	47 (21)
III	391 (16)	74 (12)	134 (18)	149 (17)	34 (16)
IV	1193 (49)	289 (47)	511 (69)	305 (35)	88 (40)
Treatment					
Surgery	484 (20)	232 (37)	55 (7)	110 (13)	87 (40)
Surgery plus aRT	444 (18)	146 (24)	97 (13)	162 (19)	39 (18)
Surgery plus CRT	307 (13)	89 (14)	135 (18)	60 (7)	23 (11)
Surgery plus chemotherapy	9 (0)	2 (0)	1 (0)	6 (1)	0 (0)
Chemo only	82 (3)	25 (4)	25 (3)	30 (3)	2 (1)
Radiation only	366 (15)	24 (4)	72 (10)	251 (29)	19 (9)
CRT, no surgery	656 (27)	69 (11)	329 (44)	213 (25)	45 (21)
No treatment	101 (4)	34 (5)	30 (4)	33 (4)	4 (2)

a Brazilian: nationality, due lack of race/ethnicity information for the Sao Paulo study. aRT = adjuvant radiotherapy; Col = column; CRT = chemoradiotherapy; NOS = not otherwise specified.

b Others: American Indian or Alaska Native.

Most (35%) tumors were hypopharyngeal and/or laryngeal, followed by oropharyngeal (30%) and oral (25%). Approximately two-thirds (65%) of patient cases were late-stage (III and IV), and 51% of reported treatment modalities were surgery based (ie, surgery alone, surgery plus adjuvant radiotherapy, surgery plus chemoradiotherapy), and 45% were chemo- and/or radiotherapy based (ie, chemotherapy alone, radiotherapy alone, chemoradiotherapy). Surgery-based regimes were reported for 75% of oral, 39% of hypopharyngeal or laryngeal, and 38% of oropharyngeal squamous cell carcinoma patients, and most included adjuvant treatment (ie, chemotherapy, radiotherapy, or both) (Supplementary Tables 1 and 2, available online).

In terms of oral health, most patients had more than 20 natural teeth, brushed less than once daily, used mouthwash, and visited their dentist 1-5 times over the past decade (Table 2). Among patients with available data, gingival bleeding was reported by approximately one-third of patients. The number of natural teeth was associated with tumor location; patients diagnosed with oral, hypopharyngeal, and laryngeal squamous cell carcinoma had fewer natural teeth than those with tumors in other sites. Smoking status was also statistically significantly associated with the number of natural teeth; reports of at least 20 natural teeth were 44% among current and 50% among former smokers, compared with 65% among nonsmokers.

**Figure 2.**
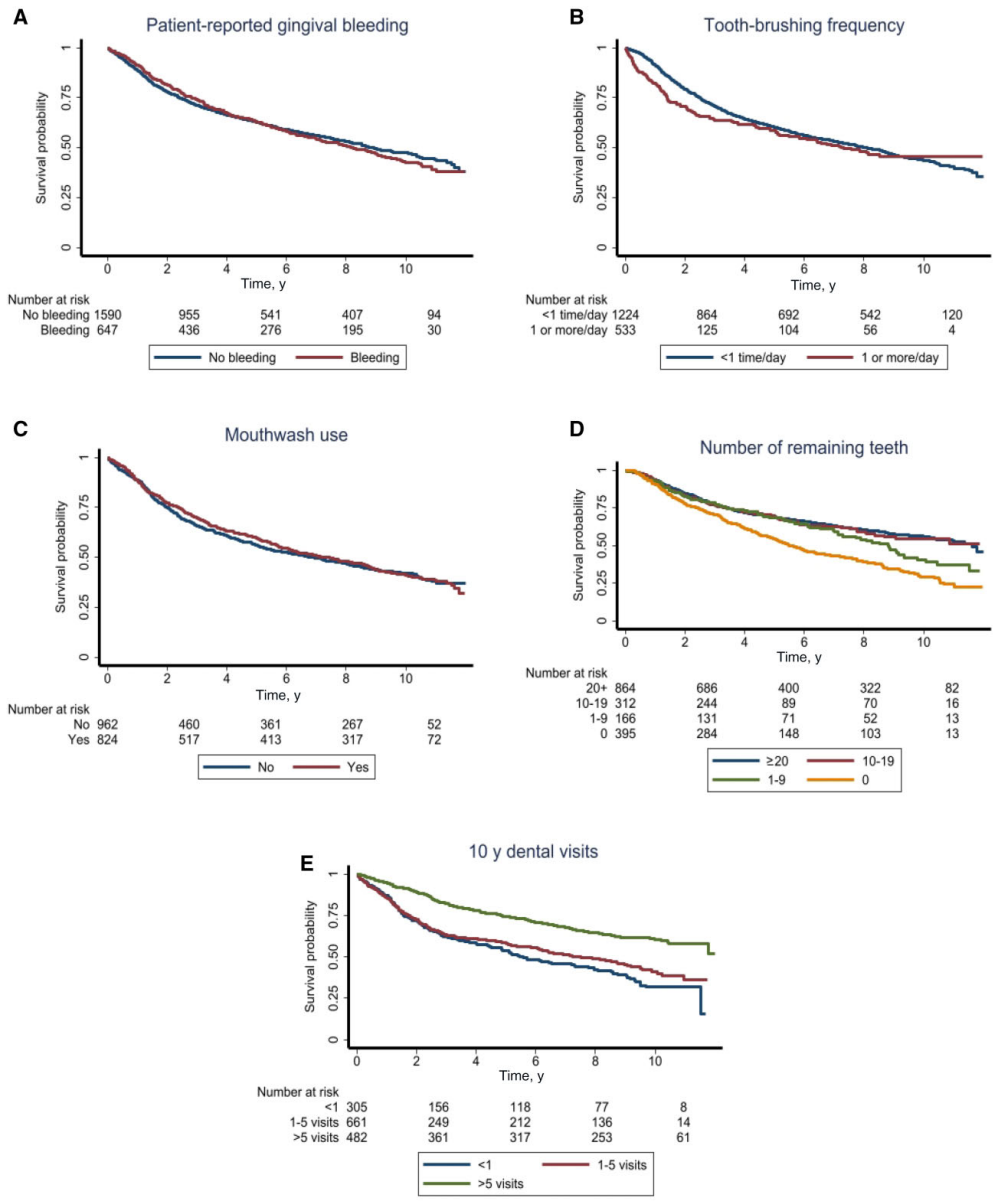
Kaplan–Meier curves for self-reported gingival bleeding **(A)**, tooth brushing **(B)**, mouthwash use **(C)**, number of natural teeth **(D)**, and number of dental visits during the past 10 years **(E).**

Comparisons of crude survival curves between strata of oral health measures revealed statistically significant associations with edentulism, maintenance of natural dentition, tooth brushing, and dental visits (Figure 2, A-E). For example, a survival benefit was found among participants who reported more than 5 dental visits during the past 10 years (5-year overall survival = 74% and 10-year overall survival = 60%) compared with those with no dental visits (5-year overall survival = 54% and 10-year overall survival = 32%) (Figure 2, D). Of note, dental visits during the past 10 years prior to diagnosis were also associated with early vs late-stage diagnosis, with the percentage of early stage patient cases diagnosed increasing from patients reporting no dental visits (21%) to patients reporting 1-5 dental visits (37%) and patients reporting more than 5 dental visits (42%) (*P* < .0005). When stratifying by site, these differences persisted only among patients with oral or hypopharyngeal and/or laryngeal head and neck squamous cell carcinoma (Table 3). Moreover, having no natural remaining teeth was associated with 15% lower 5-year overall survival compared with at least 20 natural teeth (Figure 2, E). Smaller survival differences (ie, <5%) were found for patient reported gingival bleeding, tooth brushing, and mouthwash use.

**Table 3.** Associations between dental visits during the past 10 years and early (stages I and II) vs late (stages III and IV) head and neck squamous cell carcinoma diagnosis^a^

	Dental visits during the past 10 years						
	<1		1-5		>5		
Site	Early stage (row %)	Late stage (row %)	Early stage (row %)	Late stage (row %)	Early stage (row %)	Late stage (row %)	*P*
All sites	101 (7)	204 (14)	173 (12)	488 (34)	198 (14)	284 (20)	<.0005
Oral cavity	28 (7)	58 (15)	56 (14)	169 (43)	45 (11)	36 (9)	<.0005
Oropharynx	12 (3)	56 (14)	19 (5)	149 (37)	18 (5)	144 (36)	.339
Hypopharynx and/or Larynx	50 (10)	74 (15)	84 (17)	147 (29)	87 (17)	64 (13)	<.0005
Head and neck squamous cell carcinoma NOS	11 (7)	16 (11)	14 (9)	23 (15)	48 (32)	40 (26)	.164

a NOS = not otherwise specified.

Frequent dental visits and presence of natural teeth were associated with better survival in fully adjusted multivariable analyses (Table 4). Specifically, remaining natural teeth were associated with improved survival compared with no natural teeth (eg, RR = 0.81, 95% CI = 0.69 to 0.95). Dental visits were also associated with better survival (eg, >5 visits during the last 10 years compared with none: RR = 0.77, 95% CI = 0.66 to 0.91). These associations persisted after excluding edentulous patients. Associations between natural teeth and survival were more pronounced among hypopharyngeal/laryngeal (RR = 0.75, 95% CI = 0.59 to 0.96) and NOS squamous cell carcinoma patient cases (RR = 0.66, 95% CI = 0.46 to 0.94), whereas those for dental visits were more pronounced among oropharyngeal and NOS squamous cell carcinoma (Table 4). Patient-reported gingival bleeding, tooth brushing, and mouthwash use did not show any important associations. Patients located in South America or Europe had statistically significantly decreased risk of dying compared with patients located in North America, while associations between oral health and survival were mostly similar (Supplementary Table 3, available online).

**Table 4.** Association of measures of oral health measures with overall survival estimated with a generalized linear regression model using log-link function and Poisson family regression in head and neck squamous cell carcinoma patients from the International Head and Neck Cancer Epidemiology Consortium studies

	All sites^a^	Oral cavity^b^	Oropharynx^b^	Hypopharynx and/or Larynx^b^	Head and neck squamous cell carcinoma NOS^b^
	Risk ratio (95% CI)	Risk ratio (95% CI)	Risk ratio (95% CI)	Risk ratio (95% CI)	Risk ratio (95% CI)
Patient reported gingival bleeding					
No	Referent	Referent	Referent	Referent	Referent
Yes	1.04 (0.95 to 1.14)	0.96 (0.78 to 1.18)	1.04 (0.85 to 1.28)	1.04 (0.90 to 1.20)	1.13 (0.89 to 1.45)
Tooth brushing					
<1 time daily	Referent	Referent	Referent	Referent	Referent
≥1 time(s) daily	0.90 (0.74 to 1.09)	0.88 (0.60 to 1.28)	1.05 (0.76 to 1.47)	0.80 (0.56 to 1.14)	0.88 (0.45 to 1.71)
Mouthwash					
No	Referent	Referent	Referent	Referent	Referent
Yes	1.07 (0.98 to 1.18)	1.09 (0.87 to 1.36)	1.26 (1.01 to 1.57)	1.06 (0.91 to 1.22)	0.93 (0.76 to 1.15)
Natural teeth					
0	Referent	Referent	Referent	Referent	Referent
1-9	0.90 (0.76 to 1.06)	0.95 (0.57 to 1.61)	0.83 (0.54 to 1.27)	0.82 (0.65 to 1.03)	1.29 (0.94 to 1.76)
10-19	0.81 (0.69 to 0.95)	0.93 (0.66 to 1.32)	0.80 (0.57 to 1.11)	0.75 (0.59 to 0.96)	0.66 (0.46 to 0.94)
≥20	0.88 (0.78 to 0.99)	1.11 (0.85 to 1.43)	0.83 (0.64 to 1.08)	0.87 (0.73 to 1.04)	0.66 (0.50 to 0.88)
Dental visits during the last 10 y					
0	Referent	Referent	Referent	Referent	Referent
1-5	0.95 (0.82 to 1.09)	1.10 (0.82 to 1.48)	0.77 (0.57 to 1.03)	1.01 (0.81 to 1.25)	0.71 (0.46 to 1.10)
>5	0.77 (0.66 to 0.91)	1.15 (0.78 to 1.68)	0.67 (0.49 to 0.93)	0.82 (0.64 to 1.05)	0.57 (0.38 to 0.85)

a All sites column is adjusted for age, sex, race, geographic region, tumor site, TNM stage, treatment, education, and smoking. CI = confidence intervals; NOS =  not otherwise specified; TNM = tumor-node-metastasis.

b Oral cavity, Oropharynx, Hypopharynx and/or Larynx, and head and neck squamous cell carcinoma NOS columns are stratified by tumor site and adjusted for age, sex, race, TNM stage, treatment, education, and smoking.

## Discussion

In this report, we present the results of a comprehensive analysis of the largest cohort of head and neck squamous cell carcinoma patients used to investigate the impact of oral health on survival. We found that head and neck squamous cell carcinoma patients with more than 10 natural teeth had better survival compared with those with no teeth, while those with a history of more than 5 dental visits during the past 10 years had better survival compared with those with no dental visits. These associations persisted after adjustment for potential confounders, including age, sex, race, geographic region, tumor site, smoking status, and education level with or without additional adjustments for TNM stage and treatment modality. These results identify 2 important measures of oral health—natural dentition and dental visits—as independent prognostic factors in head and neck squamous cell carcinoma. The important benefits of dental visits are further illustrated by the finding of an association between frequent dental visits and early stage head and neck squamous cell carcinoma diagnosis. Collectively, these results identify a previously overlooked role of oral health in the survival of head and neck cancer patients.

Although oral health and oral health proxies have been previously identified as risk factors in the pooled cohort of INHANCE Consortium patients (12), data regarding the role of oral health measures in head and neck squamous cell carcinoma patient survival are limited. Farquhar et al. (24) analyzed data from the CHANCE study (n = 1381 patient cases, also included in this pooled analysis) and identified strong associations between dental visits (>10 over the past decade) and overall survival (HR = 0.63, 95% CI = 0.46 to 0.89), with the association being more pronounced among patients with oral squamous cell carcinoma (HR = 0.40, 95% CI = 0.17 to 0.93). Notably, factors associated with an elevated risk, including periodontal disease, tooth mobility, and no tooth brushing were not associated with survival in this cohort. This, combined with the more pronounced effect of dental visits among patients with oral squamous cell carcinoma, indicates that dental visits are associated with survival in the context of their role as an oral health proxy and not as a health-maintenance proxy. This was further supported by the fact that this study evaluated other routine screening exams (ie, physical exams, eye exam, and colonoscopies), none of which was associated with survival. Chang et al. (23) analyzed a cohort of 740 head and neck squamous cell carcinoma patients and identified an association between a binary variable of regular dental visits and a poor oral hygiene score, defined as brushing less than twice a day, no use of dental floss, and no regular dental visits to be associated with survival. Contrary, a study of 263 head and neck squamous cell carcinoma cases by Friemel et al. (25) failed to find an association between oral hygiene score or mouthwash use and overall, disease-specific, or progression-free survival.

The small sample sizes of previous studies, the limited number of oral health measures examined, and the inconsistent associations among different studies have limited the ability to draw firm conclusions about the influence of oral health on patient survival. This is also reflected in the recent guidelines of the European Society of Medical Oncology–European Head and Neck Society–European Society for Radiotherapy and Oncology published in 2020 (30), which recommend dental screening only in the context of staging and/or prevention of radiotherapy-related adverse oral health outcomes [eg, osteonecrosis (31)]. On the other hand, the American Society for Clinical Oncology 2017 guidelines define dental care as the “diagnosis and treatment of dental caries, periodontal disease, and other intraoral conditions” and endorse this practice along with smoking cessation for patients diagnosed with head and neck squamous cell carcinoma in the context of health promotion (32). The findings of our study support this American Society for Clinical Oncology guideline by highlighting the importance of dental care, not only for the prevention of treatment-related adverse outcomes and improved quality of life but also for tertiary prevention of head and neck squamous cell carcinoma, and thus aiming to improve patient survival and ameliorate disease impact. Although the American Dental Association is endorsing screening for oral cancer and premalignant lesions on all patients (33), the current US Preventive Service Task Force recommendation regarding oral cancer screening states that the data on dental visits are insufficient to endorse it as a secondary or tertiary preventive measure (34). However, our results, and especially the associations between frequency of dental visits prior to diagnosis and early stage diagnosis with improved survival, indicate that oral examination may provide early disease detection and thus improve survival. Recent evidence from a population-representative study (35) further supports the links between oral health and cancer mortality, wherein each 10 missing permanent teeth were associated with 19% higher risk for cancer mortality among US adults.

Potential explanations for the associations between oral health and overall survival include either a mechanistic link between oral health and head and neck squamous cell carcinoma or an indirect association where oral health is a proxy for overall wellness (eg, adherence to follow-up, more frequent screening appointments). The retrospective nature of this study prohibits it from answering this, but accumulating evidence supports a link between inflammation and cancer (36). Our results also indicate an association of geographic region with survival, with patients living in South America or Europe having better survival than patients living in North America, after adjusting for other demographic, clinicopathological, treatment, and oral health variables. These associations could be attributed to several factors including racial differences, different proportions of patients from rural vs urban vs metropolitan context (37), access to health-care differences (38), different proportion of HPV-positive head and neck squamous cell carcinoma patient cases (39), and different times that each study took place (the North American cohort was established much earlier than some of the European [UK5000] and all South American cohorts).

Although our study has several strengths, there are some limitations. Not all INHANCE studies included information on all oral health measures, and definitions and measurements of these oral health parameters frequently varied. We addressed this by opting for the definition that allowed the inclusion of the largest number of patient cases. Also, most patients were missing posttreatment oral hygiene data, and thus our estimates could not be adjusted for this. In addition, alcohol consumption was available for only a small subset of the entire cohort, and thus, the variable was excluded from further analysis. The retrospective nature of all studies introduces the possibilities of recall bias and risk of exposure misclassification for some participants. However, given that any association between oral health and head and neck squamous cell carcinoma survival was probably not widely known among study participants, any potential recall bias should be minimal. Also, the evaluation of periodontal tissue status was not done clinically but rather was based on patient-reported bleeding in most studies. The absence of gingival bleeding when evaluated with a periodontal probe by a trained dentist is an indicator with very high (98%) negative predictive value for periodontal health. Although there are no data to compare patient-reported bleeding and bleeding on probing, it is reasonable to assume that a reduction on sensitivity with the former. HPV status and p16 expression were not available for most of the studies included and thus were not part of the analysis. However, most oropharyngeal squamous cell carcinoma are HPV positive, therefore it is possible that associations detected between measures of oral health and oropharyngeal squamous cell carcinoma could also be associated with HPV status. To address this limitation and avoid the potential confounding effect of not adjusting for HPV status, we performed supplementary analyses after excluding all oropharyngeal patient cases. The effect magnitude and precision were similar between analytical samples including and excluding oropharyngeal cases. Residual confounding by smoking and socioeconomic status, which is represented by educational level, remains a possibility. Also, the lack of performance status, comorbidity, and cancer treatment adherence variables could confound the results.

In conclusion, we present an analysis of the largest cohort of head and neck squamous cell carcinoma patients with oral health measures, wherein we identify strong associations between retention of natural teeth and dental visits with better survival. These results emphasize the role of oral health maintenance not only to avoid treatment-related adverse outcomes like osteoradionecrosis but also as a potentially independent prognostic parameter for head and neck squamous cell carcinoma patients. Additional prospective studies are needed to replicate and extend our findings and help elucidate mechanistic pathways at play.

## Supplementary Material

djad156_Supplementary_Data

## Data Availability

Description of the studies in the INHANCE Consortium can be found in the consortium webpage (https://medicine.utah.edu/dfpm/inhance/members/studies). Data are available from the corresponding author upon reasonable request for scientific purposes, with the permission of the INHANCE Consortium.
