# Supplementary material for: Poor oral health influences head and neck cancer patient survival: an International Head and Neck Cancer Epidemiology Consortium pooled analysis
Source: J Natl Cancer Inst. 2023 Sep 19;116(1):105–14. doi: 10.1093/jnci/djad156 (PMC10777670; doi:10.1093/jnci/djad156)
Supplement: djad156_Supplementary_Data [file djad156_supplementary_data.pdf]

Supplementary Tables

**Supplementary Table 1.** Descriptive statistics for HNSCC patients from the INHANCE Consortium studies by patient-reported gingival bleeding, tooth brushing frequency and mouthwash use.

| | **Entire sample** | **Patient-reported gingival bleeding** | | **Tooth brushing frequency** | | **Mouthwash use** | |
|---|---|---|---|---|---|---|---|
| | *N (%)* | No (%) | Yes (%) | <1 time daily (%) | 1+ time(s) daily (%) | No (%) | Yes (%) |
| Total | 2449 (100) | 1590 (100) | 647 (100) | 1224 (100) | 533 (100) | 962 (100) | 824 (100) |
| **Age, years; μ**(SD) | 59.8 (10.5) | 59 (11) | 60 (10) | 59 (10) | 58 (10) | 60 (10) | 59 (10) |
| **Sex** | | | | | | | |
| Female | 528 (22) | 330 (21) | 150 (23) | 273 (22) | 82 (15) | 177 (18) | 176 (21) |
| Male | 1921 (78) | 1260 (79) | 497 (77) | 951 (78) | 451 (85) | 785 (82) | 648 (79) |
| **Race** | | | | | | | |
| Asian & Pacific Islanders | 16 (1) | 13 (1) | 1 (0) | 0 (0) | 3 (1) | 1 (0) | 2 (0) |
| Black | 320 (13) | 228 (14) | 87 (13) | 259 (21) | 54 (10) | 118 (12) | 201 (24) |
| Brazilian | 2 (9) | 2 (0) | 0 (0) | 0 (0) | 2 (0) | 2 (0) | 0 (0) |
| Others | 138 (6) | 98 (6) | 34 (5) | 39 (3) | 86 (16) | 93 (10) | 42 (5) |
| White | 1973 (81) | 1249 (79) | 525 (81) | 926 (76) | 388 (73) | 748 (78) | 579 (70) |
| **Smoking status** | | | | | | | |
| Never smoker | 355 (14) | 260 (16) | 60 (9) | 154 (13) | 52 (10) | 103 (11) | 96 (12) |
| Former smoker | 816 (33) | 506 (32) | 216 (33) | 304 (25) | 137 (26) | 255 (27) | 206 (25) |
| Current smoker | 1278 (52) | 824 (52) | 371 (57) | 766 (63) | 344 (65) | 604 (63) | 522 (63) |
| **Education level** | | | | | | | |
| ≤Junior high school | 333 (14) | 165 (10) | 90 (14) | 105 (9) | 125 (23) | 195 (20) | 54 (7) |
| Some high school | 945 (39) | 648 (41) | 216 (33) | 341 (28) | 291 (55) | 388 (40) | 271 (33) |
| High school graduate | 466 (19) | 311 (20) | 142 (22) | 327 (27) | 66 (12) | 157 (16) | 235 (29) |
| Technical school | 431 (18) | 285 (18) | 131 (20) | 289 (24) | 40 (8) | 141 (15) | 178 (22) |
| ≥College graduate | 274 (11) | 181 (11) | 68 (11) | 162 (13) | 11 (2) | 81 (8) | 86 (10) |
| **TNM stage** | | | | | | | |
| I | 458 (19) | 282 (18) | 130 (20) | 258 (21) | 40 (8) | 148 (15) | 155 (19) |
| II | 407 (17) | 242 (15) | 126 (19) | 228 (19) | 66 (12) | 153 (16) | 143 (17) |
| III | 391 (16) | 261 (16) | 101 (16) | 211 (17) | 79 (15) | 160 (17) | 137 (17) |
| IV | 1193 (49) | 805 (51) | 290 (45) | 527 (43) | 348 (65) | 501 (52) | 389 (47) |
| **Tumor site** | | | | | | | |
| Oral cavity | 621 (25) | 386 (24) | 159 (25) | 200 (16) | 202 (38) | 249 (26) | 169 (21) |
| Oropharynx | 744 (30) | 512 (32) | 175 (27) | 330 (27) | 141 (26) | 263 (27) | 211 (26) |
| Hypopharynx /Larynx | 865 (35) | 541 (34) | 246 (38) | 486 (40) | 180 (34) | 364 (38) | 313 (38) |
| HNSCC NOS | 219 (9) | 151 (9) | 67 (10) | 208 (17) | 10 (2) | 86 (9) | 131 (16) |
| **Treatment** | | | | | | | |
| Surgery | 484 (20) | 341 (21) | 115 (18) | 264 (22) | 73 (14) | 173 (18) | 174 (21) |
| Surgery+aRT | 444 (18) | 303 (19) | 121 (19) | 258 (21) | 102 (19) | 180 (19) | 187 (23) |

| | | | | | | | |
|---|---|---|---|---|---|---|---|
| Surgery+CRT | 307 (13) | 215 (14) | 73 (11) | 163 (13) | 78 (15) | 128 (13) | 117 (14) |
| Surgery+Chemo | 9 (0) | 4 (0) | 3 (0) | 3 (0) | 4 (1) | 7 (1) | 2 (0) |
| Chemo only | 82 (3) | 40 (3) | 24 (4) | 15 (1) | 48 (9) | 54 (6) | 2 (0) |
| Radiation only | 366 (15) | 228 (14) | 108 (17) | 197 (16) | 46 (9) | 120 (12) | 129 (16) |
| CRT, no surgery | 656 (27) | 404 (25) | 182 (28) | 303 (25) | 128 (24) | 240 (25) | 202 (25) |
| No treatment | 101 (4) | 55 (3) | 21 (3) | 21 (2) | 54 (10) | 60 (6) | 11 (1) |

HNSCC NOS: Head and Neck Squamous Cell Carcinoma Not otherwise specified; aRT: Adjuvant radiotherapy.

**Supplementary Table 2.** Descriptive statistics for HNSCC patients from the INHANCE Consortium studies by missing teeth and dental visits during the past 10 years.

| | Entire sample | Natural teeth | | | | Dental visits during the past 10 years | | |
|---|---|---|---|---|---|---|---|---|
| | *N* (%) | 0 (%) | 1-9 (%) | 10-19 (%) | 20+ (%) | 0 (%) | 1-5 (%) | >5 (%) |
| **Total** | 2449 (100) | 395 (100) | 166 (100) | 312 (100) | 864 (100) | 305 (100) | 661 (100) | 482 (100) |
| **Age**, years; mean (SD) | 59.8 (10.5) | 63 (9) | 63 (9) | 61 (9) | 58 (11) | 60 (10) | 59 (10) | 58 (11) |
| **Sex** | | | | | | | | |
| Female | 528 (22) | 107 (27) | 42 (25) | 69 (22) | 190 (22) | 66 (22) | 123 (19) | 112 (23) |
| Male | 1921 (78) | 288 (73) | 124 (75) | 243 (78) | 674 (78) | 239 (78) | 538 (81) | 370 (77) |
| **Race** | | | | | | | | |
| White | 1973 (81) | 314 (79) | 134 (81) | 262 (84) | 717 (83) | 242 (79) | 493 (75) | 438 (91) |
| Black | 320 (13) | 74 (19) | 28 (17) | 45 (14) | 123 (14) | 42 (14) | 77 (12) | 25 (5) |
| Asian & Pacific Islanders | 16 (1) | 1 (0) | 0 (0) | 4 (1) | 8 (1) | 0 (0) | 1 (0) | 2 (0) |
| Others | 138 (6) | 6 (2) | 4 (2) | 1 (0) | 16 (2) | 21 (7) | 89 (13) | 16 (3) |
| Brazilian | 2 (9) | 0 (0) | 0 (0) | 0 (0) | 0 (0) | 0 (0) | 1 (0) | 1 (0) |
| **Smoking status** | | | | | | | | |
| Never smoker | 355 (14) | 33 (8) | 19 (11) | 50 (16) | 188 (22) | 21 (7) | 68 (10) | 108 (22) |
| Former smoker | 816 (33) | 120 (30) | 59 (36) | 129 (41) | 314 (36) | 74 (24) | 177 (27) | 156 (32) |
| Current smoker | 1278 (52) | 242 (61) | 88 (53) | 133 (43) | 362 (42) | 210 (69) | 416 (63) | 218 (45) |
| **Education level** | | | | | | | | |
| ≤Junior high school | 333 (14) | 47 (12) | 13 (8) | 11 (4) | 33 (4) | 57 (19) | 163 (25) | 30 (6) |
| Some high school | 945 (39) | 158 (40) | 66 (40) | 114 (37) | 244 (28) | 137 (45) | 290 (44) | 68 (14) |
| High school graduate | 466 (19) | 83 (21) | 35 (21) | 77 (25) | 204 (24) | 58 (19) | 90 (14) | 113 (23) |
| Technical school | 431 (18) | 68 (17) | 40 (24) | 67 (21) | 213 (25) | 42 (14) | 82 (12) | 148 (31) |
| ≥College graduate | 274 (11) | 39 (10) | 12 (7) | 43 (14) | 170 (20) | 11 (4) | 36 (5) | 123 (26) |
| **TNM stage** | | | | | | | | |
| I | 458 (19) | 89 (23) | 39 (23 | 73 (23) | 190 (22) | 43 (14) | 90 (14) | 116 (24) |
| II | 407 (17) | 83 (21) | 41 (25) | 52 (17) | 140 (16) | 58 (19) | 83 (13) | 82 (17) |
| III | 391 (16) | 67 (17) | 22 (13) | 54 (17) | 137 (16) | 49 (16) | 100 (15) | 82 (17) |
| IV | 1193 (49) | 156 (39) | 64 (39) | 133 (43) | 397 (46) | 155 (51) | 388 (59) | 202 (42) |
| **Tumor site** | | | | | | | | |
| Oral cavity | 621 (25) | 83 (21) | 28 (17) | 64 (12) | 157 (18) | 86 (28) | 225 (34) | 81 (17) |
| Oropharynx | 744 (30) | 90 (23) | 46 (28) | 119 (38) | 313 (36) | 68 (22) | 168 (25) | 162 (34) |
| Hypopharynx /Larynx | 865 (35) | 180 (46) | 77 (46) | 106 (34) | 261 (30) | 124 (41) | 231 (35) | 151 (31) |

| | | | | | | | | |
|---|---|---|---|---|---|---|---|---|
| HNSCC NOS | 219 (9) | 42 (11) | 15 (9) | 23 (7) | 133 (15) | 27 (9) | 37 (6) | 88 (18) |
| **Treatment** | | | | | | | | |
| Surgery | 484 (20) | 84 (21) | 34 (20) | 71 (23) | 203 (23) | 56 (18) | 89 (13) | 117 (24) |
| Surgery+aRT | 444 (18) | 89 (23) | 34 (20) | 53 (17) | 154 (18) | 61 (20) | 107 (16) | 102 (21) |
| Surgery+CRT | 307 (13) | 39 (10) | 14 (8) | 35 (11) | 126 (15) | 40 (13) | 90 (14) | 73 (15) |
| Surgery+Chemo | 9 (0) | 0 (0) | 0 (0) | 0 (0) | 2 (0) | 2 (1) | 5 (1) | 2 (0) |
| Chemo only | 82 (3) | 0 (0) | 2 (1) | 0 (0) | 2 (0) | 20 (7) | 49 (7) | 11 (2) |
| Radiation only | 366 (15) | 86 (22) | 36 (22) | 65 (21) | 128 (15) | 39 (13) | 77 (12) | 56 (12) |
| CRT, no surgery | 656 (27) | 95 (24) | 45 (27) | 88 (28) | 246 (28) | 67 (22) | 179 (27) | 108 (22) |
| No treatment | 101 (4) | 2 (1) | 1 (1) | 0 (0) | 3 (0) | 20 (7) | 65 (10) | 13 (3) |

HNSCC NOS: Head and Neck Squamous Cell Carcinoma Not otherwise specified; aRT: Adjuvant radiotherapy.

**Supplementary Table 3**. Association of measures of oral health measures with overall survival estimated with a generalized linear regression model using log link function and Poisson Family regression in HNSCC patients from the INHANCE Consortium studies. All sites column is adjusted for age, sex, race, geographic region, tumor site, TNM stage, treatment, education, and smoking. Europe, North America and South America columns are adjusted for age, sex, race, tumor site, TNM stage, treatment, education, and smoking.

| **Geographic region** | **All regions** | **Europe** | **North America** | **South America** |
|---|---|---|---|---|
| | RR 95% CI | RR 95% CI | RR 95% CI | RR 95% CI |
| **Patient reported gingival bleeding** | | | | |
| No | Ref. | *Ref.* | Ref. | *Ref.* |
| Yes | 1.04 (0.95-1.14) | 1.18 (0.92-1.51) | 0.98 (0.89-1.09) | 0.92 (0.66-1.29) |
| **Tooth brushing** | | | | |
| <1 time daily | Ref. | *Ref.* | Ref. | *Ref.* |
| 1+ time(s) daily | 0.90 (0.74-1.09) | 1.21 (0.86-1.71) | 0.93 (0.71-1.21) | 0.72 (0.50-1.03) |
| **Mouthwash** | | | | |
| No | Ref. | *Ref.* | Ref. | *Ref.* |
| Yes | 1.07 (0.98-1.18) | NE | 1.06 (0.96-1.16) | 1.23 (0.93-1.63) |
| **Natural teeth** | | | | |
| 0 | Ref. | *Ref.* | Ref. | *Ref.* |
| 1-9 | 0.90 (0.76-1.06) | 0.97 (0.55-1.70) | 0.89 (0.76-1.05) | NE |
| 10-19 | 0.81 (0.69-0.95) | 0.89 (0.56-1.40) | 0.83 (0.70-0.97) | NE |
| 20+ | 0.88 (0.78-0.99) | 1.05 (0.68-1.60) | 0.88 (0.78-0.99) | NE |
| **Dental visits during the last 10 years** | | | | |
| 0 | Ref. | *Ref.* | Ref. | *Ref.* |
| 1-5 | 0.95 (0.82-1.09) | 1.11 (0.82-1.50) | 0.98 (0.82-1.17) | 0.83 (0.62-1.10) |
| >5 | 0.77 (0.66-0.91) | 0.93 (0.54-1.61) | 0.83 (0.70-0.99) | 0.48 (0.27-0.88) |

RR: Risk Ratio; CI; Confidence Intervals; Ref.: Reference; HNSCC NOS: Head and Neck Squamous Cell Carcinoma Not otherwise specified.
